# Supplementary material for: The diagnostic value of pleural fluid homocysteine in malignant pleural effusion
Source: PLoS One. 2019 Sep 24;14(9):e0222616. doi: 10.1371/journal.pone.0222616 (PMC6759144; doi:10.1371/journal.pone.0222616)
Supplement: S1 Table — (DOC) [file pone.0222616.s001.doc]

**S1 Table. Descriptive statistics of independent variables (n=133).**

| **Biomarker** | **Range** | **Median (95% CI)** | **IQR** | **Distribution*** |
| --- | --- | --- | --- | --- |
| **Edad** (years) | 1-96 | 70 (65-72) | 21 | Reject normality (p<0.0001) |
| **HCY** (µmol/L) | 1.82-34.30 | 10.70 (9.77-11.69) | 5.49 | Reject normality (p<0.0001) |
| **CEA** (ng/mL) | 0.20-4207 | 1.39 (0.93-2.08) | 5.24 | Reject normality (p<0.0001) |
| **CA15.3** (U/mL) | 1.0-300 | 11.6 (9.6-15.3) | 22.3 | Reject normality (p<0.0001) |
| **CA19.9** (U/mL) | 0.6-4059 | 2.2 (1.3-2.9) | 6.4 | Reject normality (p<0.0001) |
| **CA125** (U/mL) | 2.8-11599 | 522 (437-621) | 728 | Reject normality (p<0.0001) |

HCY: homocysteine; CEA: carcinoembryonic antigen; CA: cancer antigen; CI: confidence interval; IQR: interquartile range; *D'Agostino-Pearson test for normal distribution.
