# Supplementary material for: The diagnostic value of pleural fluid homocysteine in malignant pleural effusion
Source: PLoS One. 2019 Sep 24;14(9):e0222616. doi: 10.1371/journal.pone.0222616 (PMC6759144; doi:10.1371/journal.pone.0222616)
Supplement: S2 Table — Difference between AUCs. (DOC) [file pone.0222616.s002.doc]

**S2 Table. Comparison of ROC curves. Difference between AUCs.**

|  | **CEA**  **(AUC=0.778)** | **CA15.3**  **(AUC=0.800)** | **CA19.9**  **(AUC=0.703)** | **CA125**  **(AUC=0.606)** | **HCY+CEA**  **(AUC=0.948)** |
| --- | --- | --- | --- | --- | --- |
| **HCY**  **(AUC=0.846)** | p>0.05 | p>0.05 | 0.143  (p=0.016) | 0.240  (p=0.0001) | 0.103  (p=0.002) |
| **CEA**  **(AUC=0.778)** | - | p>0.05 | p>0.05 | 0.173  (p=0.006) | 0.170  (p=0.0001) |
| **CA15.3**  **(AUC=0.800)** | - | - | p>0.05 | 0.194  (p=0.001) | 0.149  (p=0.0001) |
| **CA19.9**  **(AUC=0.703)** | - | - | - | p>0.05 | 0.246  (p=0.0001) |
| **CA125**  **(AUC=0.606)** | - | - | - | - | 0.343  (p=0.0001) |

AUC: area under the curve; CEA: carcinoembryonic antigen; CA: cancer antigen; HCY: homocysteine; HCY+CEA: probabilistic model (%) = 100 x (1 + e^-z^)^-1^; Z = 0.5471 x [HCY] + 0.3846 x [CEA] – 8.2671.
